# An Odorant-Binding Protein Is Abundantly Expressed in the Nose and in the Seminal Fluid of the Rabbit

**DOI:** 10.1371/journal.pone.0111932

**Published:** 2014-11-12

**Authors:** Rosa Mastrogiacomo, Chiara D′Ambrosio, Alberto Niccolini, Andrea Serra, Angelo Gazzano, Andrea Scaloni, Paolo Pelosi

**Affiliations:** 1 Department of Agriculture, Food and Environment, University of Pisa, Pisa, Italy; 2 Proteomics & Mass Spectrometry Laboratory, ISPAAM, National Research Council, Napoli, Italy; 3 Department of Veterinary Sciences, University of Pisa, Pisa, Italy; CNR, Italy

## Abstract

We have purified an abundant lipocalin from the seminal fluid of the rabbit, which shows significant similarity with the sub-class of pheromone carriers “urinary” and “salivary” and presents an N-terminal sequence identical with that of an odorant-binding protein (rabOBP3) expressed in the nasal tissue of the same species. This protein is synthesised in the prostate and found in the seminal fluid, but not in sperm cells. The same protein is also expressed in the nasal epithelium of both sexes, but is completely absent in female reproductive organs. It presents four cysteines, among which two are arranged to form a disulphide bridge, and is glycosylated. This is the first report of an OBP identified at the protein level in the seminal fluid of a vertebrate species. The protein purified from seminal fluid is bound to some organic chemicals whose structure is currently under investigation. We reasonably speculate that, like urinary and salivary proteins reported in other species of mammals, this lipocalin performs a dual role, as carrier of semiochemicals in the seminal fluid and as detector of chemical signals in the nose.

## Introduction

Odorant-binding proteins (OBPs) of vertebrates are a sub-class of lipocalins [1]–[2], a protein super-family including retinol-binding protein [3], ß-lactoglobulin [4] and many other members that differ for amino acid sequence and physiological function but share the highly conserved structure of the ß-barrel, a sort of cup made of 8 antiparallel ß-sheets enclosing a binding cavity for hydrophobic ligands [5]-[10]. Vertebrate OBPs are binding proteins of about 150–160 amino acids firstly identified in the nasal epithelium of mammals and classified as carriers for odorants and pheromones [11]–[17]. Several members of this family have been isolated from different mammals, such as bovine, pig, rabbit and others [18]–[25], as well as in amphibians [26]. OBPs bind to a large variety of small organic molecules, including odorants and pheromones, with a broad specificity and dissociation constants in the micromolar range [9], [27]–[31].

Despite the detailed structural and functional information available for several OBPs, their physiological role in olfaction is still not clear [15]–[17], [32]–[33]. A carrier for hydrophobic odorants across the aqueous nasal mucus seems reasonable, but a more specific function in detecting chemical messengers cannot be excluded. This idea is based on the expression of several OBPs in the same species, with different and complementary spectra of binding [30], [34]. Moreover, there is clear evidence that insect OBPs, a class of proteins structurally different from those of vertebrates, but probably with similar functions [35], are often required for a correct detection of odors and pheromones [36]–[37], and are also involved in the discrimination of different semiochemicals [38]–[39].

Whatever their role and detailed mechanism of action, it is reasonable to hypothesise that OBPs from vertebrates might be involved in the detection of pheromones, rather than general odorants. This idea is suggested by the small number of OBP sub-types reported in mammals, as compared to those from insects, and their expression in the vomeronasal organ (an organ dedicated to pheromone perception) [40]–[42] or in glands of the nasal respiratory epithelium [43], but not in the olfactory mucosa. The sole exception of the human OBP, which was detected in the mucus of the olfactory cleft, but not in the lower nasal regions [44], might be explained with the fact that the vomeronasal organ is absent or non-functioning in humans. However, strong evidence for the involvement of OBPs in detecting pheromones comes from their expression in organs dedicated to the synthesis and the delivery of pheromones [33]. In fact, OBPs similar or identical to those identified in the nose have also been reported as expressed in non-sensory organs and secreted in biological fluids involved in pheromonal communication. Best studied examples include the “major urinary proteins” (MUPs) of mouse and rat [7], [45]–[48], which are synthesised in the liver and excreted in the urine at concentrations of several mg/mL, the “salivary proteins” (SALs) of the boar, abundantly produced by the submaxillary glands [10], [19], [34], and the so-called “aphrodisin” identified in the vaginal secretion of the hamster [49]–[50]. In each species, these proteins are produced in the above-mentioned organs in a sex-specific fashion, while they are expressed in the nose equally in both sexes [51]. When released in the urine, saliva or other secretions, such proteins are loaded with organic compounds known to be the species-specific pheromones, while in the nose they are void. In particular, it has been reported that murine MUPs, when excreted in the urine, are complexed with known animal pheromones, such as 2-sec-butylthiazoline and 3,4-dehydro-*exo*-brevicomin [47], [52]. Similarly, pig SALs, when isolated from the saliva, carries the boar-specific pheromones 5α-androst-16-en-3-one and 5α-androst-16-en-3-ol [19].

Although the few cases reported above have been studied in detail, the use of OBPs as carriers of pheromones to be released in the environment might be much more common and widespread. The sweath of horses contains large amounts of an OBP-like protein complexed with putative semiochemicals [25], while the salivary lipocalins of several mammals, often reported as allergens [53]–[55], might perform similar functions. Chemical communication in the rabbit has not been widely studied. A single pheromone has been so far described, namely the volatile compound 2-methyl-2-butenal, which was isolated from the milk and shown to trigger a very clear and robust response in the puppies [56]–[57]. Information on rabbit OBPs is limited to our previous work reporting the isolation and partial characterization of three members from the nasal tissue [18], [23]. The present study was aimed at further investigating the putative role of rabbit OBPs as carriers of pheromones to be released in the environment and describes an OBP expressed only in the nose of both sexes and in seminal fluid.

## Experimental Procedures

### Materials

Rabbit bodies were kindly provided by a local abbattoir and dissected within an hour after death or kept at −20°C for a few days. Rabbit seminal fluid was collected using an all-glass artificial vagina equipped with a jacket where warm water was circulated.

### Ethics statement

All operations were carried out in strict accordance with the recommendations for handling laboratory animals of the National Research Council (CNR) of Italy. The protocol was approved by the Committee on the Ethics of Animal Experiments of the Italian CNR (Permit Number: 01-2014 of February 18, 2014). All efforts were made to minimize suffering of the animals.

### RNA extraction and cDNA synthesis

Total RNA was extracted using TRI Reagent (Sigma), following the manufacturer's protocol. cDNA was prepared from total RNA by reverse transcription, using 200 units of SuperScript™ III Reverse Transcriptase (Invitrogen) and 0.5 mg of an oligo-dT primer in a 50 µL reaction volume. The mixture also contained 0.5 mM of each dNTP (GE-Healthcare), 75 mM KCl, 3 mM MgCl2, 10 mM DTT and 0.1 mg/ml BSA in 50 mM Tris-HCl, pH 8.3. The reaction mixture was incubated at 50°C for 60 min and the product was directly used for PCR amplification or stored at −20°C.

### Polymerase chain reaction

Aliquots of 1 µL of crude cDNA were amplified in a Bio-Rad Gene Cycler thermocycler, using 2.5 units of *Thermus aquaticus* DNA polymerase (GE-Healthcare), 1 mM of each dNTP (GE-Healthcare), 1 µM of each PCR primer, 50 mM KCl, 2.5 mM MgCl_2_ and 0.1 mg/ml BSA in 10 mM Tris-HCl, pH 8.3, containing 0.1% v/v Triton X-100. At the 5′ end, we used a specific primer (rabOBP3-fw: 5′-CACAGCCACTCGGA-3′) corresponding to the sequence encoding the first five amino acids of the mature protein. At the 3′ end, we used an oligo-dT to first obtain the correct sequence of the gene, then a specific primer (rabOBP3-rv: 5′-TTAGGCGGCTCCGCCGTC-3′) encoding the last five residues and the stop codon, to check the presence of the gene in different tissues. After a first denaturation step at 95°C for 5 min, we performed 35 amplification cycles (1 min, at 95°C; 30 sec, at 50°C; 1 min, at 72°C) followed by a final step of 7 min, at 72°C.

### Cloning and sequencing

The crude PCR products were ligated into a pGEM (Promega) vector without further purification, using a 1∶5 (plasmid∶insert) molar ratio and incubating the mixture overnight, at room temperature. After transformation of *E. coli* XL-1 Blue competent cells with the ligation products, positive colonies were selected by PCR using the plasmid's primers SP6 and T7 and grown in LB/ampicillin medium. DNA was extracted using the Plasmid MiniPrep Kit (Euroclone) and custom sequenced at Eurofins MWG (Martinsried, Germany).

### Preparation of the tissue extracts

Crude extracts were prepared by homogenization of the corresponding tissues in 10 mL of 20 mM Tris-HCl pH 7.4 (Tris buffer) per gram of tissue, using a Polytron homogenizer, followed by centrifugation at 20,000×*g* for 20 min. The clear supernatant was immediately used for SDS-PAGE and Western blotting experiments.

### Purification of the seminal protein

Lipocalins from rabbit seminal fluid were purified through a 1×30 cm Superose 12 column in 50 mM ammonium bicarbonate, as previously reported [23]. Selected fractions were then pooled, dialysed against 20 mM Tris-HCl, pH 7.4, and applied to a 1.5×25 cm Whatman DE-52 column. Elution was performed using a linear 0.1–0.4 M NaCl gradient, in 20 mM Tris-HCl, pH 7.4. Each fraction was analysed using 12% SDS-PAGE.

### Protein digestion and peptide separation

Rabbit seminal fluid OBP was resolved by SDS-PAGE, excised from the gel, triturated, *in-gel* reduced, S-alkylated and digested with trypsin, as previously reported [56]. Gel particles were extracted with 25 mM NH_4_HCO_3_/acetonitrile (1∶1 v/v) by sonication, and digests were concentrated. Peptide mixtures were either desalted using μZipTipC_18_ pipette tips (Millipore) before MALDI-TOF-MS analysis, directly analyzed by nanoLC-ESI-LIT-MS/MS (see below) or simply resolved on an Easy C_18_ column (100×0.075 mm, 3 µm) (Proxeon) using a linear gradient of acetonitrile containing 0.1% trifluoroacetic acid in aqueous 0.1% trifluoroacetic acid, at a flow rate of 300 nL/min, for 80 min. In the latter case, collected fractions were concentrated and analyzed by MALDI-TOF-MS.

### Protein alkylation under native conditions

Protein samples for disulfide assignment were alkylated with 1.1 M iodoacetamide in 0.25 M Tris-HCl, 1.25 mM EDTA, and 6 M guanidinium chloride, pH 7.0, at 25 °C for 1 min in the dark. Samples were separated from excess salts and reagents by passing the reaction mixture through a PD10 column (Amersham Biosciences), as previously reported [59]. Protein samples were finally digested and resolved by LC as mentioned above.

### Glycopeptide enrichment

To isolate glycopeptides, rabbit seminal fluid OBP digest aliquots were solved in 80% acetonitrile, 2% formic acid and loaded on GELoader tips (Eppendorf, Germany), which were plugged with 3M Empore C8 extraction disk material (3M Bioanalytical Technologies, MN) and packed with ZIC-HILIC (200 Å, 10 µm, zwitterionic sulfobetaine functional groups) resin (Sequant, Sweden) [60]. Loaded microcolumns were washed twice with 15 µL of 80% acetonitrile, 2% formic acid. Glycopeptides were first eluted with 10 µL of 2% formic acid and then with 5 µL of 50% acetonitrile, 2% formic acid; pooled fractions were analyzed by MALDI-TOF-MS, as described below.

### Peptide deglycosylation and disulfide reduction

Glycopeptides were directly deglycosylated on the MALDI target by treatment with 0.2 U of PNGase F (Roche) in 50 mM NH_4_HCO_3_, pH 8, at 37 °C, for 1 h. Then, 2 µL of 0.1% trifluoroacetic acid was added to reaction mixtures, which were desalted on μZipTipC18 pipette tips (Millipore) before MALDI-TOF-TOF-MS analysis [61].

Disulfide-containing peptides were directly reduced on the MALDI target by treatment with 10 mM mM DTT in 50 mM NH_4_HCO_3_, pH 8, at 37 °C, for 1 h. Then, 2 µL of 0.1% trifluoroacetic acid was added to reaction mixtures, which were desalted on μZipTipC18 pipette tips (Millipore) before MALDI-TOF-TOF-MS analysis [61].

### MS analysis

Peptide mixtures were analyzed by nLC-ESI-LIT-MS/MS using a LTQ XL mass spectrometer (ThermoFinnigan, USA) equipped with a Proxeon nanospray source connected to an Easy-nLC (Proxeon, Denmark) [58]. They were resolved on an Easy C_18_ column (100×0.075 mm, 3 µm) (Proxeon) using a linear gradient of acetonitrile containing 0.1% formic acid in aqueous 0.1% formic acid, at a flow rate of 300 nL/min, for 25 min. Spectra were acquired in the range *m/z* 400–1800. Acquisition was controlled by a data-dependent product ion scanning procedure over the 3 most abundant ions, enabling dynamic exclusion (repeat count 1 and exclusion duration 1 min). The mass isolation window and collision energy were set to *m/z* 3 and 35%, respectively.

During MALDI-TOF-MS analysis, entire protein digests or selected peptide fractions were loaded on the instrument target together with 2,5-dihydroxy-benzoic acid (10 mg/mL in 70% v/v acetonitrile, 0.1% v/v trifluoroacetic acid) or α-cyano-4-hydroxycinnamic acid (saturated solution in 30% v/v acetonitrile, 0.1% v/v trifluoroacetic acid) as matrices, using the dried droplet technique; a 384-spot ground steel plate (Bruker Daltonics) was used to this purpose. Spectra were acquired in the *m/z* range 500–5000 on a Bruker Ultraflextreme MALDI-TOF-TOF instrument (Bruker Daltonics) operating either in reflectron mode or linear mode. Instrument settings were: pulsed ion extraction  = 100 ns, laser frequency  = 1000 Hz, number of shots per sample  = 2500–5000 (random walk, 500 shots per raster spot). Mass spectra were calibrated externally using nearest neighbour positions loaded with Peptide Calibration Standard II (Bruker Daltonics), with quadratic calibration curves. MS/MS spectra were acquired in LIFT mode. Data were elaborated using the FlexAnalysis software (Bruker Daltonics).

nLC-ESI-LIT-MS/MS data were searched by using MASCOT (version 2.2.06) (Matrix Science, UK) against an updated rabbit EST database containing available protein sequences (NCBI 28/11/2013, 212376 sequences). As searching parameters, we used a mass tolerance value of 2 Da for precursor ion and 0.8 Da for ion fragments, trypsin trypsin and/or slymotrypsin (cleavage at Lys, Arg, Phe, Tyr, Trp and Leu) as proteolytic enzymes, a missed cleavages maximum value of 2, Cys carbamidomethylation and Met oxidation as fixed and variable modification, respectively. Protein candidates with more than 2 assigned unique peptides with an individual Mascot ion score >25 and a significant threshold (*p*<0.05) were further considered for protein identification. In the case of glycopeptides or disulfide-containing peptides, MALDI-TOF mass signals were assigned to peptides, glycopeptides or disulfide-containing peptides using the GPMAW 4.23 software (Lighthouse Data, Denmark). This software generated a mass/fragment database output based on protein sequence, protease selectivity, nature of the amino acids susceptible to eventual glycosylation/oxidation and the molecular mass of the modifying groups. Searching parameters were set as mentioned above; mass values were matched to protein regions using a 0.02% mass tolerance value. MALDI-TOF-TOF searching parameters were set with tolerances of 100 ppm and 0.5 Da for MS and MS/MS data, respectively. Glycosylation or disulfide assignments were always confirmed by additional MS experiments on deglycosylated or reduced peptides, respectively.

### Ligand-binding experiments

The affinity of the fluorescent probe N-phenyl-1-naphthylamine (1-NPN) was measured by titrating a 2 µM solution of the protein with aliquots of 1 mM 1-NPN solved in methanol to reach final concentrations of 2–16 µM. The probe was excited at 337 nm and the maximum emission wavelength was 415 nm. Dissociation constant was evaluated using GraphPad Prism software. Affinities of other ligands were measured in competitive binding assays, by titrating a solution containing the protein and 1-NPN both at the concentration of 4 µM with 1 mM solutions of each competitor in methanol to reach final concentrations of 0–16 µM. Dissociation constants of the competitors were calculated from the concentrations of ligand halving the initial fluorescence value of 1-NPN (IC_50_), using the equation:
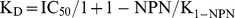



1-NPN being the free concentration of 1-NPN and K_1-NPN_ being the dissociation constant of the complex protein/1-NPN.

## Results

### Identification and purification of an OBP from the rabbit seminal fluid

**Figure 1:**
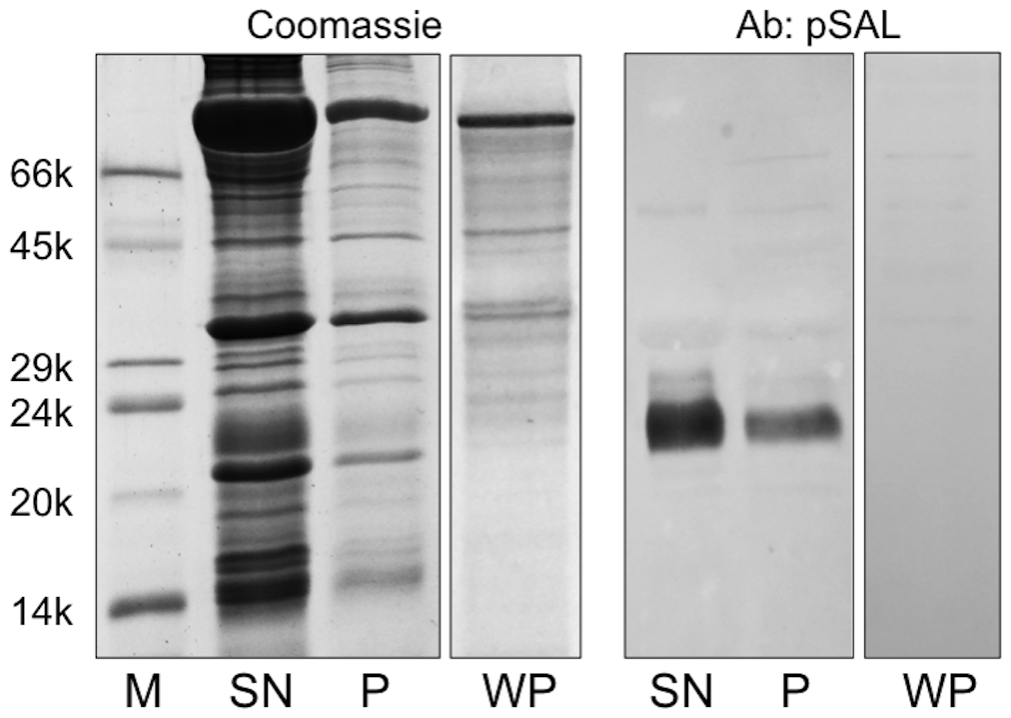
SDS-PAGE analysis of rabbit sperm and corresponding Western blotting. SN, soluble fraction; P, sperm cells; WP, sperm cells after washing three times with buffer. A strong cross-reactivity with a polyclonal antiserum raised against pig SAL [19] was observed for a protein migrating at about 23 kDa. Staining was much stronger in the soluble fraction; the weak reactivity observed for the sperm cells disappeared after washing the cells, thus indicating the absence of the protein in this sample.

**Figure 2:**
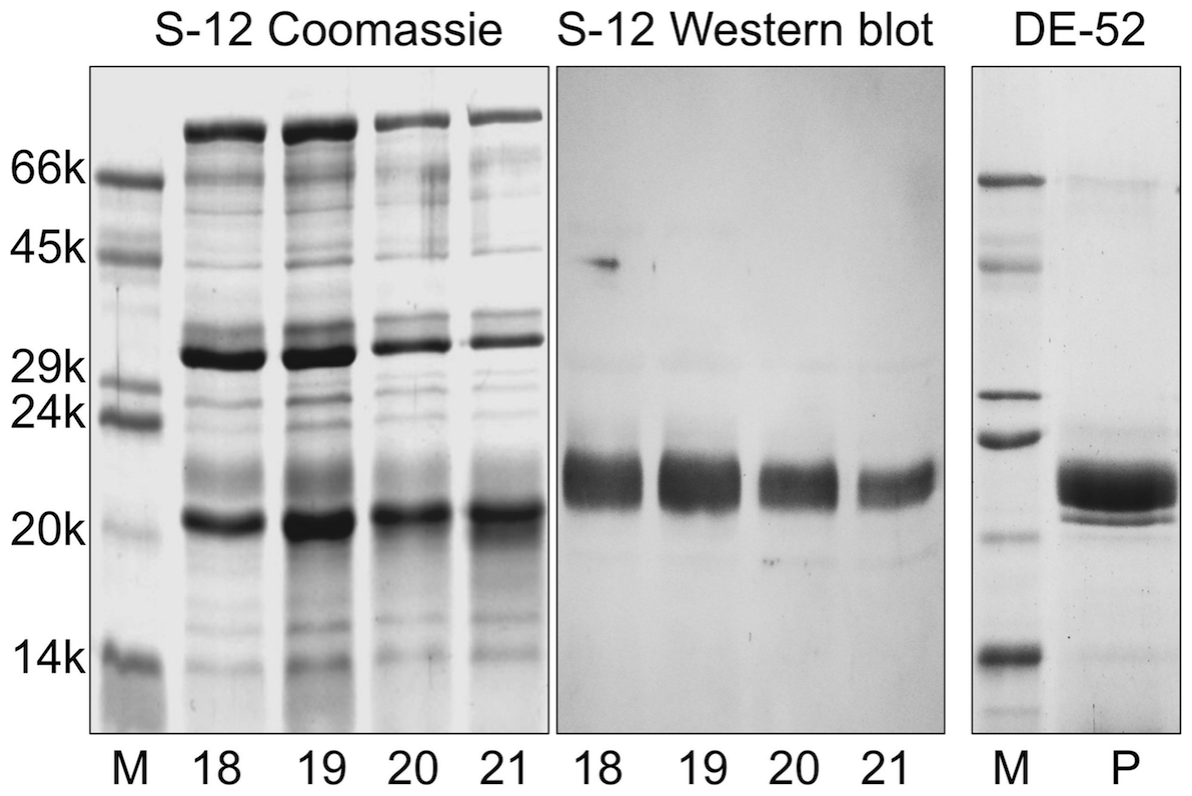
Purification of the rabbit seminal fluid OBP. A sample of crude seminal fluid, as obtained after sperm centrifugation, was resolved at first by gel filtration chromatography on a Superose-12 column and then by anion-exchange chromatography on a DE-52 column (see Materials and Methods section for details). The protein was eluted as a pure component, as verified by SDS-PAGE.

With the aim of identifying OBPs expressed in rabbit non-sensory organs, we verified the occurrence of a protein in the male semen that showed a cross-reactivity with a polyclonal antiserum raised against the boar salivary lipocalin (pig SAL) [19]. This protein, which migrated in SDS-PAGE as a blurred band at about 23 kDa, was very abundant in the seminal liquid but was not present in the sperm cells. Figure 1 reports the electrophoretic analysis of the supernatant and the pellet obtained by centrifugation of the crude semen. The weaker cross-reactivity of the pellet was due to a contamination with the seminal fluid and disappeared completely after washing the pellet three times with buffer. Protein concentration in the semen was estimated to be about 10–20 mg/mL. This protein was then purified by gel filtration chromatography on a Superose-12 column, followed by anion-exchange chromatography on a DE-52 resin. Figure 2 reports the SDS-PAGE profile of selected fractions from the first purification step, together with the corresponding Western blotting, as well as of the purified protein that was used for further studies.

In order to characterize the nature of this seminal protein, we performed a MALDI-TOF peptide mass fingerprinting analysis on its tryptic digest following reduction with dithiothreitol and alkylation with iodoacetamide (data not shown). MS results matched to a sequence reported in the NCBI EST database (entry EL341998) annotated as UTE-7, which corresponded to a cDNA isolated from rabbit uterus. The sequence at the protein N-terminus of UTE-7 is identical with that of a rabbit OBP (rabOBP3) we had previously isolated from the nasal tissue [23]. Since the identity of some nucleotides in the EST entry mentioned above was not determined and the sequence was partial, we again cloned the corresponding cDNA and sequenced it; data are reported in Supplementary Figure S1. Our analysis provided a complete nucleotide assignment, together with very few base corrections, finally ascertaining a corresponding protein sequence as made of 161 amino acids. Finally, massive peptide mapping nanoLC-ESI-LI-MS/MS experiments on a tryptic digest ascertained the nature of the protein N- and C-terminus, verifying about 93% of its amino acid sequence (Table S1).

### Tissue expression

**Figure 3:**
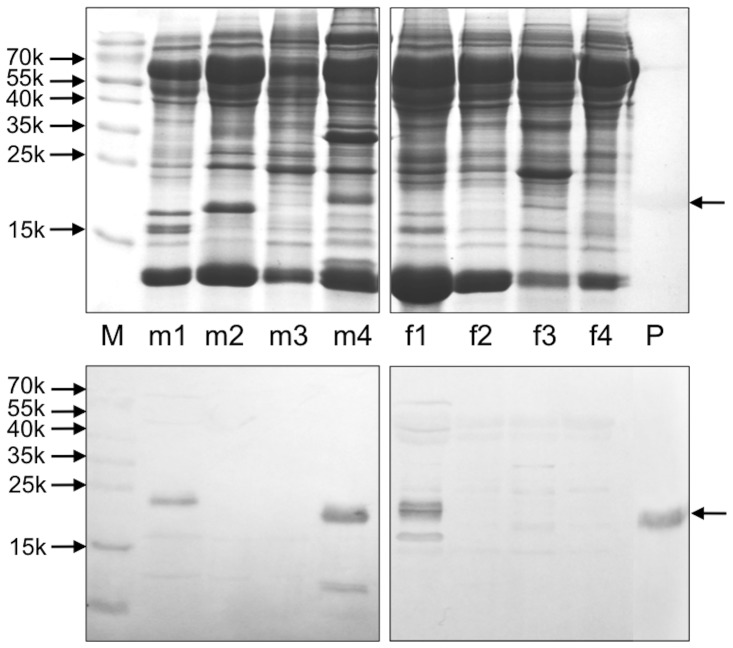
Expression of rabOBP3 in different tissues of male (m) and female (f) rabbit individuals. SDS-PAGE analysis of different rabbit tissues and corresponding Western blotting are shown. M: molecular weight markers; m1: nasal respiratory tissue; m2: epidydimis; m3: testis; m4: prostate; f1: nasal respiratory tissue; f2: uterine tubes; f3: ovaries; f4: uterus; P: purified rabOBP3.

To detect the site of synthesis for this seminal protein, we performed PCR experiments on samples of cDNA prepared from different parts of male and female reproductive organs. To first identify the full sequence of the gene (Figure S1), we used a specific primer at the 5′-end encoding the first five amino acids of the sequence reported in the database as UTE-7 (acc. no: EL341998) and an oligo-dT at the 3′-end. Then, we used the same primer at the 5′-end and a second specific primer at the 3′-end encoding the last five residues and the stop codon, to check for the presence of this gene in different organs. In particular, olfactory and respiratory epithelium from both sexes, prostate, epididymis, testis, uterus, uterine tubes, ovaries, vagina and vaginal vestibule were evaluated. Amplification bands were obtained only for the prostate as well as for the respiratory epithelium of both sexes. Parallel cloning and sequencing of samples from these tissues always yielded the same sequence (Figure S1), excluding the occurrence of various protein isoforms. The specificity of protein expression in these tissues was confirmed at the protein level by Western-blotting experiments (Figure 3). On this basis, we can conclude that the protein previously named as UTE-7 is not produced in the uterus, nor in any part of the female reproductive system, but was probably found in such organ as result of a sample contamination. On the other hand, the sequence we report here very likely corresponds to the protein (rabOBP3) we had previously isolated from the nasal epithelium [23]. Accordingly, we decided to rename UTE-7 as rabOBP3.

### Post-translational modifications in rabOBP3

**Figure 4:**
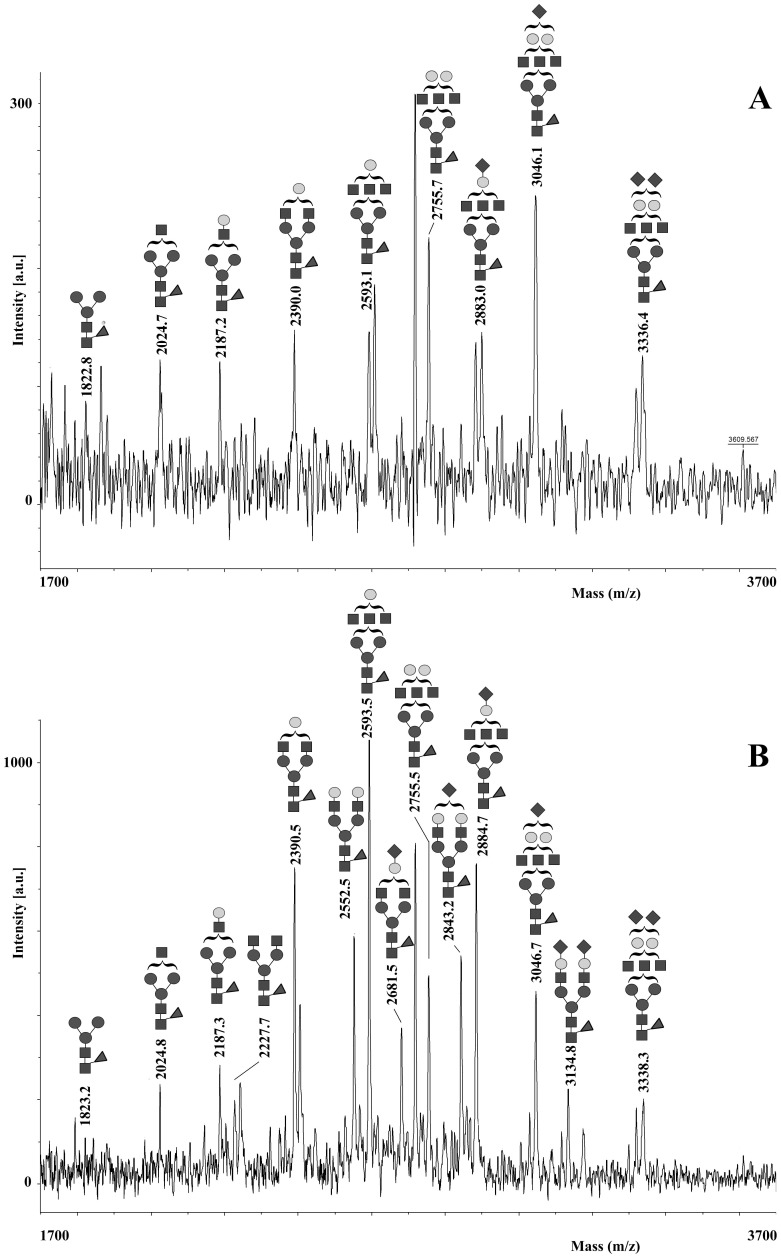
MALDI-TOF-MS analysis of the purified tryptic glycopeptides from rabOBP3 as obtained after HILIC enrichment and nanoLC separation. Spectra acquired in linear mode of the fractions eluting at 15 and 16 min are reported in panel A and B, respectively; shown are the mono-, bi- and tri-antennary complex-type glycan structures N-linked to Asn44 in peptide (44–50). ▪, N-acetyl-glucosamine; •, mannose; ○, galactose; ◂, fucose; ♦, N-acetyl-neuraminic acid.

The blurred band and the discrepancy between the calculated (18 kDa) and apparent (23 kDa) molecular mass of the intact protein observed in SDS-PAGE, its broad MH^+^ signal in MALDI-TOF-MS (data not shown) and the occurrence of two putative N-linked glycosylation sites (Asn29 and Asn44) in the corresponding amino acid sequence (as predicted by bioinformatic analysis) suggested that rabOBP3 could be a glycoprotein, similarly to what reported for pig SAL, horse EquC1 and some murine/rat MUPs [25], [34], [62]. To evaluate protein glycosylation and assign potential modification site(s), a rabOBP3 sample resolved by SDS-PAGE was *in gel* reduced, alkylated with iodoacetamide and digested with trypsin. The corresponding peptide digest was then enriched for glycopeptides on a HILIC column and resolved by nanoLC into different fractions, which were then analyzed by MALDI-TOF-MS. Fractions eluting at 15 and 16 min showed a similar pattern of multiple signals in the mass spectrum (Figure 4A and B). On the basis of the measured mass values and known pathways of glycoprotein biosynthesis, all these peaks were assigned to peptide (44–50) having a pentasaccharide core N-linked to Asn44, and bearing mono-, bi- and tri-antennary complex glycan structures (theor. MH^+^ values: *m/z* 1821.8, 2024.9, 2187.1, 2228.2, 2390.3, 2552.5, 2593.5, 2681.6, 2755.7, 2843.7, 2884.8, 3046.9, 3135.0 and 3338.2). After PNGase treatment, glycopeptides in both fractions collapsed to a unique component (peptide 44–50) having a MH^+^ signal at *m/z* 784.08 (data not shown). MALDI-TOF-TOF-MS analysis of the deglycosylated peptide confirmed the expected Asn44>Asp conversion. Multiple signals associated with glycopeptides were also detected in the mass spectrum of the fractions eluting at 21 and 22 min. On the basis of measured mass values (exp. MH^+^ values: *m/z* 3124.8, 3327.9, 3490.2, 3530.9, 3693.2, 3733.5, 3855.4, 3896.6, 3983.8, 4059.1, 4146.4, 4187.7, 4350.1, 4437.0, 4641.2 and 4932.9) and the relative intensities, these peaks were associated to peptide (34–50) having the same glycan structures reported in Figure 4 as N-linked to Asn44 (theor. MH^+^ values: *m/z* 3123.3, 3326.5, 3488.7, 3529.7, 3691.9, 3732.9, 3854.0, 3895.0, 3983.1, 4057.2, 4145.3, 4186.3, 4348.4, 4436.5, 4639.7 and 4931.0). No signals related to the non-glycosylated peptide counterparts were detected in any LC fractions either from the entire protein digest or its glycopeptide-enriched portion, thus suggesting that rabOBP3 was completely modified at this site. On the other hand, no glycopeptides containing the other putative N-linked glycosylation site (Asn29) were observed in the tryptic digest or its HILIC eluate either before and after nanoLC separation; conversely, the corresponding non-glycosylated counterparts were always detected in both cases, thus demonstrating that no modification occurred at this site.

**Figure 5:**
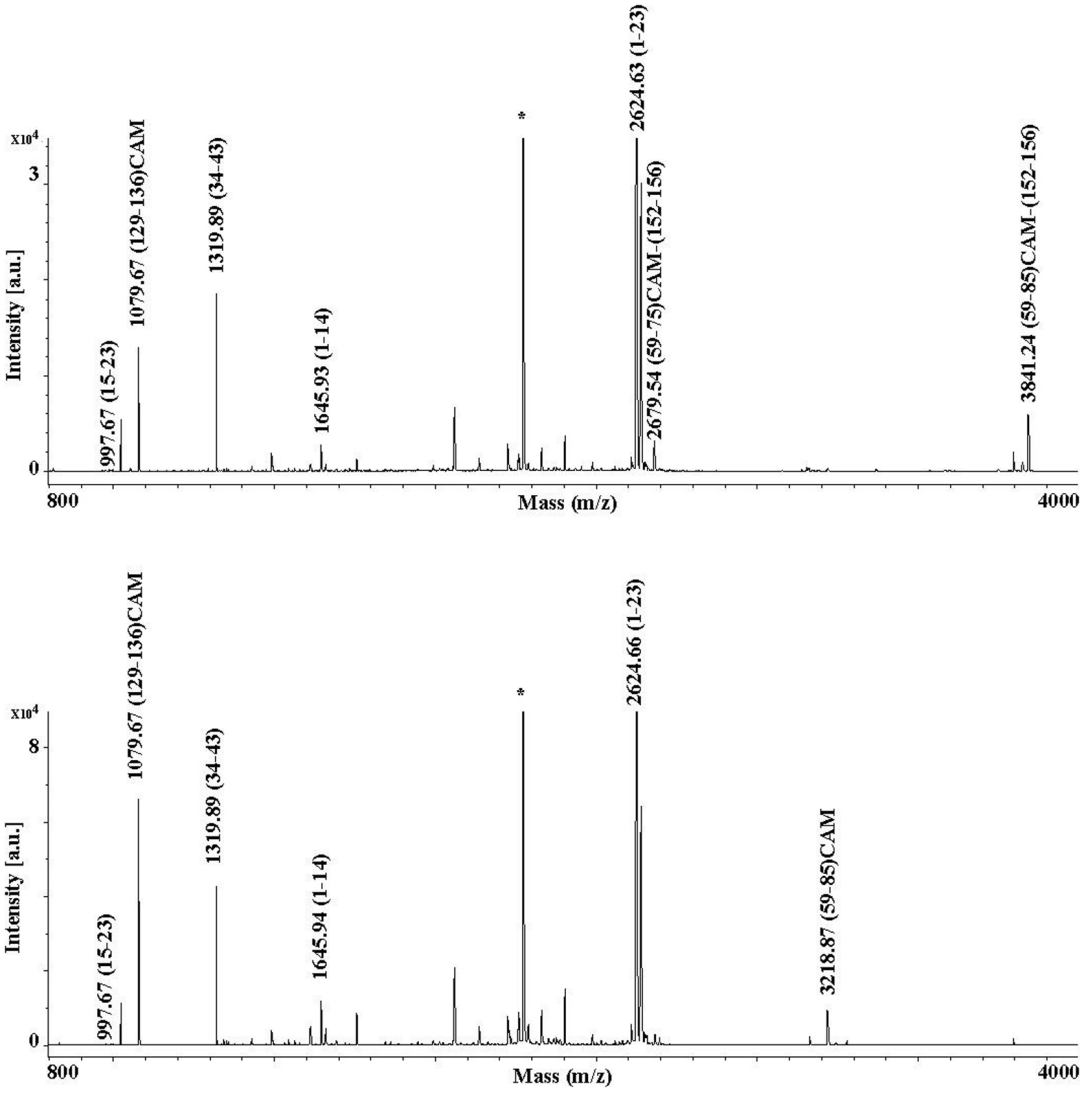
MALDI-TOF-MS analysis of the tryptic digest of rabOBP3 alkylated with iodoacetamide under denaturing, non-reducing conditions before (top) and following (bottom) treatment with dithiothreitol. Constant and variable signals are labelled in the spectra acquired in reflectron mode to highlight reduced and oxidized residues present under native conditions. Trypsin-derived peptides are indicated with an asterisk.

To evaluate protein thiol status and assign disulfide-bridged Cys residues, if present, rabOBP3 was treated with 1.1 M iodoacetamide under denaturing, non-reducing conditions and purified by size-exclusion chromatography. The alkylated protein was then digested with trypsin and split in two samples that were treated or not with DTT; Figure 5 shows the MALDI-TOF mass spectrum of each sample. In addition to a number of common signals present in both spectra, the digest deriving from the protein not treated with DTT uniquely showed the presence of a clear MH^+^ signal at *m/z* 3841.24, which was associated with the disulfide-containing peptides (59–85)CAM-(152–156) resulting from an aspecific cleavage at Phe85. A faint MH^+^ peak at *m/z* 2679.54 was also observed; this signal was assigned to the smaller disulfide-containing peptide homologue (59–75)CAM-(152–156) derived from an aspecific hydrolytic event at Tyr85. Conversely, the digest treated with DTT showed the absence of the signals mentioned above and the exclusive occurrence of a MH^+^ peak at *m/z* 3218.87, which was associated with the peptide (59–85)CAM. Due to its reduced mass value, no signal assigned to the peptide (152–156) was observed. These result confirmed the occurrence of one cysteine (Cys59 or Cys66) involved in a disulfide bond with Cys152 in the above-mentioned peptides, the remaining one being in a reduced status. On the other hand, both samples showed the presence of a MH^+^ signal at *m/z* 1079.67, which derived from the peptide (129–136)CAM; the latter result demonstrate that rabOBP3 contains Cys133 as free thiol under native conditions.

**Figure 6:**
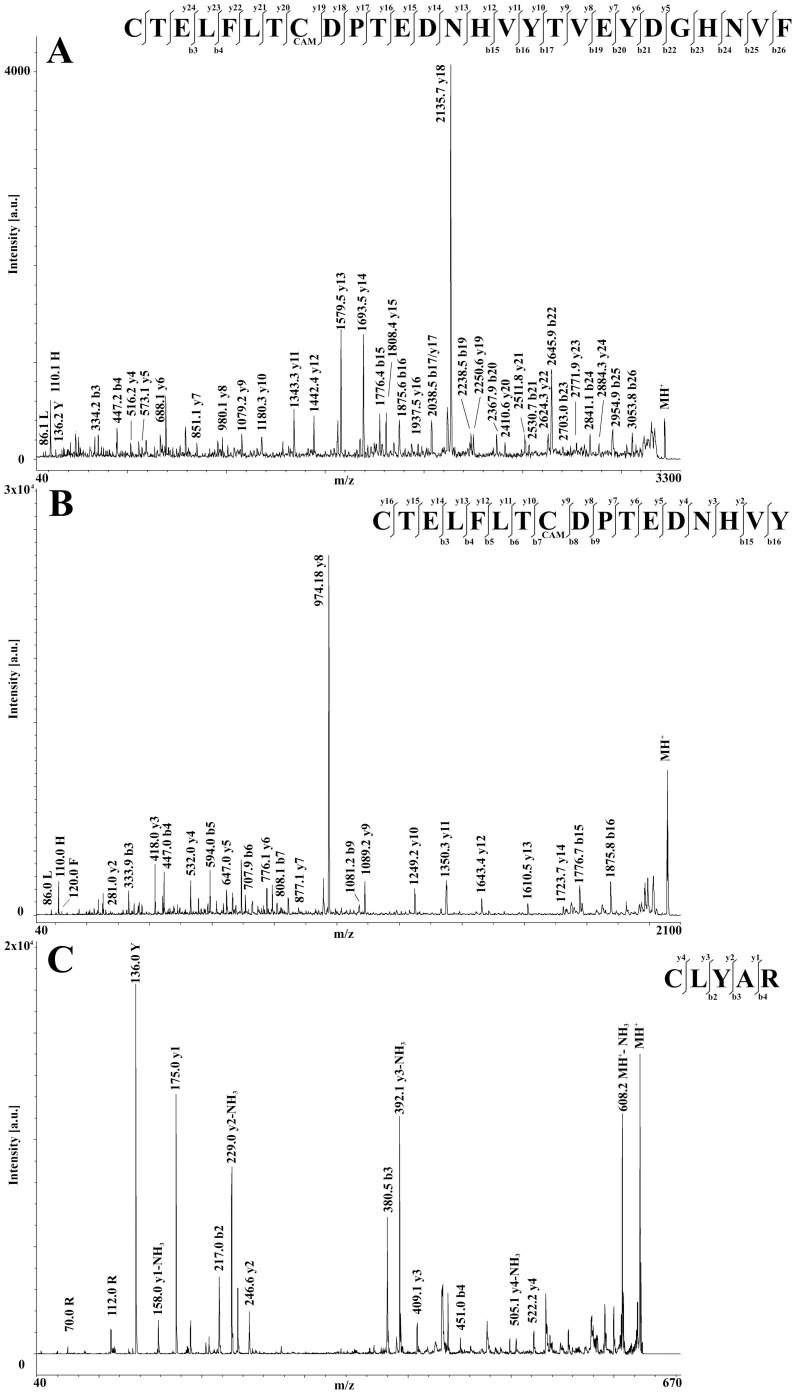
MALDI-TOF-TOF spectra of the disulfide-containing tryptic peptides from alkylated rabOBP3 following treatment with dithiothreitol. Fragmentation spectra of the peptides (59–85)CAM, (59–75)CAM and (152–156) are shown in panels A, B and C, respectively. In all cases, Cys residues originally involved in the S-S bond are present in a reduced status, the remaining ones occurring as carboxamidomethylated derivatives.

To definitively assign the Cys residues involved in the protein S-S bond, disulfide-containing peptides (59–85)CAM-(152–156) and (59–75)CAM-(152–156) were then purified by nanoLC and reduced with DTT directly on the MALDI target. Resulting products showed MH^+^ peaks at *m/z* 3220.2 and 2058.6, which were associated with the expected reduced peptides (59–85)CAM and (59–75)CAM, respectively, both having the Cys residue originally involved in the S-S bond in a reduced status and the remaining one as carboxamidomethylated species. In both cases, the occurrence of the reduced peptide (152–156) was also observed in the corresponding MS spectra (exp. MH^+^ signal at *m/z* 625.2). MALDI-TOF-TOF-MS analysis of the reduced peptides (59–85)CAM and (59–75)CAM finally assigned the thiol group to Cys59, definitively proving the existence of a disulfide bond in rabOBP3 linking together Cys59 and Cys152 (Figure 6).

### Endogenous ligands of rabOBP3

Since pig SAL and murine/rat MUPs carry species-specific pheromones as endogenous ligands, we then searched for compounds that might be complexed with rabOBP3. Gas-chromatographic separation coupled with MS (GC-MS) analysis of a dichloromethane extract of the protein from rabbit seminal liquid showed the presence of several peaks, to none of which we could confidently assign a defined chemical structure.

**Figure 7:**
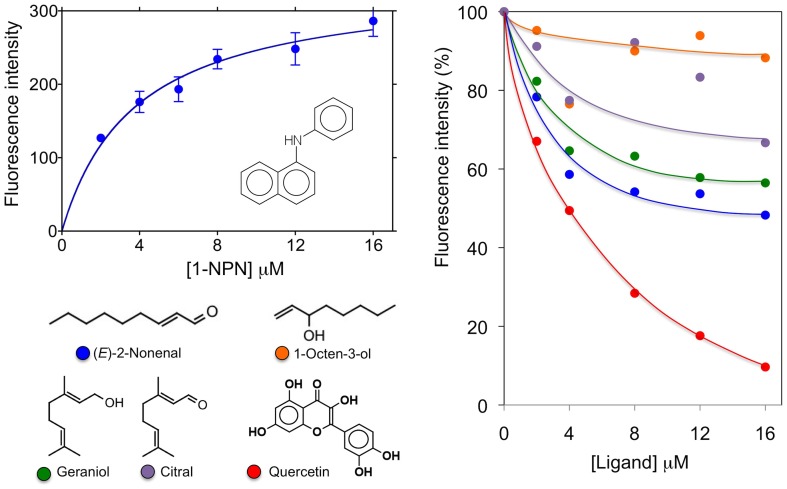
Binding of 1-NPN (left) and selected ligands (right) to rabOBP3 purified from seminal fluid and delipidated with dichloromethane. The protein binds the fluorescent probe 1-NPN with a dissociation constant of 3.8 µM (SD 0.9, n = 3). None of the ligands tested exhibited strong affinity to the protein, except quercetin, for which a physiological role does not seem plausible. Calculated dissociation constants are 2.2, 7.8 and 11.2 µM for quercetin, 2-nonenal and geraniol, respectively.

Ligand-binding assays showed that rabOBP3 reversibly binds to the fluorescent probe N-phenyl-1-naphthylamine (1-NPN) with a dissociation constant of 3.8 µM (SD 0.9, n = 3). Competitive binding assays, performed with some common plant volatiles indicated significant, but modest affinity to 2-nonenal and geraniol. On the other hand, quercetin efficiently displaced 1-NPN from the complex, but is difficult to propose a role as a rabbit semiochemical for this compound (Figure 7).

### Three-dimensional model of rabOBP3

**Figure 8:**
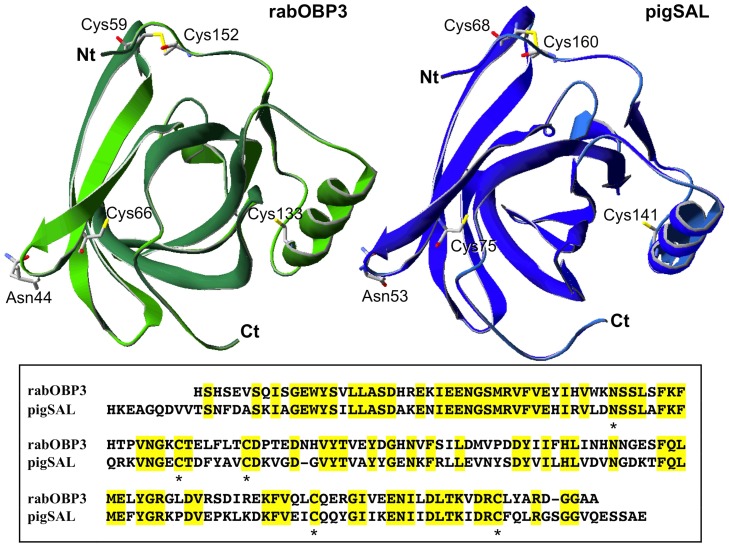
Three-dimensional model of rabOBP3 as built by using the crystallographic structure of pig SAL (Boar salivary lipocalin, PDB ID: 1 GM6) as a template[10]. Molecular model of rabOBP3 and pig SAL are shown in the left- and right-top panel, respectively. The corresponding sequence alignment is shown in the bottom panel, where conserved amino acids are highlighted in yellow. The conserved N-glycosylation site (Asn44), and oxidized (Cys59 and Cys152) and reduced (Cys66 and Cys133) residues are indicated by specific labelling (top) or asterisks (bottom).

Based on the significant (52%) sequence identity between rabOBP3 and pig SAL (Figure 8, bottom), a three-dimensional molecular model of the first protein was built up as deriving from the crystal structure of the latter (Boar salivary lipocalin, PDB ID: 1 GM6) (Figure 8, top). The good quality of this model was assessed by ANOLEA and GROMOS evaluations, which calculated small positive energy values for very few amino acids scattered along the sequence. Although not fixed as initial structural constrains before the modelling procedure, a *post hoc* evaluation of the rabOBP3 model was in perfect agreement with the protein post-translational modifications determined in this study. In fact, Asn44 occurred at the most external position in a loop extending its side chain into the solvent, while Cys59 and Cys152 were present in the model with their S atoms at a distance compatible with the presence of a disulfide bridge (Figure 8). The latter result was not surprising, based on the high conservation of cystine moieties in rabOBP3, pig SAL, murine/rat MUPs, and other proteins [49]–[63]. As expected, the remaining cysteine residues (Cys66 and Cys133) occurred too far apart to be linked together, in a condition compatible with a reduced state.

## Discussion

When the first OBP of vertebrates was discovered in the nasal tissue of the cow [11]–[12], its sequence similarity with urinary proteins of rodents immediately suggested a function in chemical communication for these polypeptides [64], which had been described several years earlier, but whose presence in the urine had represented an unsolved puzzle until then [65]–[66]. Since that time, the occurrence of proteins of the same class or even identical in olfactory organs and in secretions used in chemical communication has been well documented both in vertebrates and in insects. These polypeptides can be recognised among the family of OBPs on the basis of sequence similarity. Besides the urinary proteins of mouse and rat, OBPs of vertebrates include the boar salivary lipocalin SAL [30], the horse Equc1 (abundantly secreted in sweat) [25] and the hamster aphrodisin occurring in the vaginal discharge [49]. On the other hand, the human genome contains a pseudogene for a protein of this group, which presents a mutation at the donor site of the second intron, thus disrupting the corresponding ORF [67].

Insects OBPs have been reported in the sex organs. In particular, mosquito *Aedes aegypti* and lepidopteran *Helicoverpa armigera* OBPs, which also occur in the insect antennae, are produced in the male reproductive organ and are transferred to the female during mating. It has been shown that *H. armigera* OBP, when extracted from semen, is complexed with potential pheromones for the species and eventually is found on the surface of fertilised eggs [68]. In vertebrates, OBPs have been reported in reproductive organs: aphrodisin is secreted in the vaginal discharge of the hamster [47]–[48], while in humans the gene encoding an OBP is expressed in the prostate [69]. Data reported in this study suggest that also in the seminal liquid of the rabbit, OBPs might act as pheromone carriers. Unfortunately, information on rabbit pheromones is limited to the suckling pheromone, which directs pups towards the nipple [56]–[57]. Among the volatiles we have extracted from seminal rabOBP3, we were not able to identify any compound with confidence, thus suggesting that endogenous ligands of rabOBP3 might not be among common natural chemicals. In line with this consideration, preliminary competitive binding assays with common terpenoids and fatty acids excluded these compounds as protein endogenous ligands.

In conclusion, we propose that OBPs as pheromone carriers are likely present in the seminal fluid of other mammals. The isolation of OBPs in reproductive organs and the identification of their endogenous ligands could lead to the discovery of novel pheromones mediating behaviour between sexes, such as male competition, in mammals as it has been shown in some insect species. Besides the knowledge advancement in the biology of mammals, such information might suggest strategies to improve rearing conditions of economically important species, such as rabbit, cattle, pigs and horses.

## Supporting Information

Figure S1 (**A**) PCR amplification of the gene encoding rabOBP3 in the prostate (P), as well as in male (mR) and female (fR) nasal respiratory tissue. All three samples gave amplification bands of around 500 bp, that were cloned and sequenced yielding the same sequence, reported in (**B**) with its translation. Similar experiments performed in the same conditions on uterus (Ut), uterine tubes (Tb) and ovaries (Ov) did not produce any amplification bands. (**C**) Alignment of the derived mature amino acid sequences of rabOBP3 cloned from nose and prostate, and compared with the sequence stored in the NCBI EST database as UTE-7 (entry EL341998). Mnose: male nasal tissue; Fnose: female nasal tissue; Prost: prostate.
(TIF)

Table S1 **Results of a peptide mapping nanoLC-ESI-LI-MS/MS experiment on a tryptic digest of rabbit seminal OBP.**
(XLSX)

## References

[1] Flower DR (1996) The lipocalin protein family: structure and function. Biochem J 318: 1–14. 8761444 10.1042/bj3180001 PMC1217580

[2] Flower DR (2000) Experimentally determined lipocalin structures. Biochim Biophys Acta 1482: 46–56. 11058746 10.1016/s0167-4838(00)00147-3

[3] Monaco HL, Rizzi M, Coda A (1995) Structure of a complex of two plasma proteins: transthyretin and retinol-binding protein. Science 268: 1039–1041. 7754382 10.1126/science.7754382

[4] Sawyer L, Kontopidis G (2000) The core lipocalin ß-lactoglobulin. Biochim Biophys Acta 1482: 136–148. 11058756 10.1016/s0167-4838(00)00160-6

[5] Bianchet MA, Bains G, Pelosi P, Pevsner J, Snyder SH, et al (1996) The three dimensional structure of bovine odorant-binding protein and its mechanism of odor recognition. Nat Struct Biol 3: 934–939. 8901871 10.1038/nsb1196-934

[6] Tegoni M, Ramoni R, Bignetti E, Spinelli S, Cambillau C (1996) Domain swapping creates a third putative combining site in bovine odorant binding protein dimer. Nat Struct Biol 3: 863–867. 8836103 10.1038/nsb1096-863

[7] Böcskei Z, Groom CR, Flower DR, Wright CE, Phillips EV, et al (1992) Pheromone binding to two rodent urinary proteins revealed by X-ray crystallography. Nature 360: 186–188. 1279439 10.1038/360186a0

[8] Spinelli S, Ramoni R, Grolli S, Bonicel J, Cambillau C, et al (1998) The structure of the monomeric porcine odorant binding protein sheds light on the domain swapping mechanism. Biochemistry 37: 7913–7918. 9609684 10.1021/bi980179e

[9] Vincent F, Spinelli S, Ramoni R, Grolli S, Pelosi P, et al (2000) Complexes of porcine odorant binding protein with odorant molecules belonging to different chemical classes. J Mol Biol 300: 127–139. 10864504 10.1006/jmbi.2000.3820

[10] Spinelli S, Vincent F, Pelosi P, Tegoni M, Cambillau C (2002) Boar Salivary Lipocalin: Three-dimensional X-Ray Structure and Androstenol/Androstenone Docking Simulations. Eur J Biochem 269: 2449–2456. 12027882 10.1046/j.1432-1033.2002.02901.x

[11] Pelosi P, Pisanelli AM, Baldaccini NE, Gagliardo A (1981) Binding of 3H-2-isobutyl-3-methoxypyrazine to cow olfactory mucosa. Chem Senses 6: 77–85.

[12] Pelosi P, Baldaccini NE, Pisanelli AM (1982) Identification of a specific olfactory receptor for 2-isobutyl-3-methoxypyrazine. Biochem J 201: 245–248. 7082286 10.1042/bj2010245 PMC1163633

[13] Bignetti E, Cavaggioni A, Pelosi P, Persaud KC, Sorbi RT, et al (1985) Purification and characterization of an odorant binding protein from cow nasal tissue. Eur J Biochem 149: 227–231. 3996407 10.1111/j.1432-1033.1985.tb08916.x

[14] Pevsner J, Trifiletti RR, Strittmatter SM, Snyder SH (1985) Isolation and characterization of an olfactory receptor protein for odorant pyrazines. Proc Natl Acad Sci USA 82: 3050–3054. 2986147 10.1073/pnas.82.9.3050 PMC397704

[15] Pelosi P (1994) Odorant-binding proteins. Crit Rev Biochem Mol Biol 29: 199–228. 8070277 10.3109/10409239409086801

[16] Pelosi P (1996) Perireceptor events in olfaction. J Neurobiol 30: 3–19. 8727979 10.1002/(SICI)1097-4695(199605)30:1<3::AID-NEU2>3.0.CO;2-A

[17] Tegoni M, Pelosi P, Vincent F, Spinelli S, Campanacci V, et al (2000) Mammalian odorant binding proteins. Biochim Biophys Acta 1482: 229–240. 11058764 10.1016/s0167-4838(00)00167-9

[18] Dal Monte M, Andreini I, Revoltella R, Pelosi P (1991) Purification and characterization of two odorant binding proteins from nasal tissue of rabbit and pig. Comp Biochem Physiol 99B: 445–451. 10.1016/0305-0491(91)90068-o 1764925

[19] Marchese S, Pes D, Scaloni A, Carbone V, Pelosi P (1998) Lipocalins of boar salivary glands binding odours and pheromones. Eur J Biochem 252: 563–568. 9546674 10.1046/j.1432-1327.1998.2520563.x

[20] Pes D, Mameli M, Andreini I, Krieger J, Weber M, et al (1998) Cloning and expression of odorant-binding proteins Ia and Ib from mouse nasal tissue. Gene 212: 49–55. 9661663 10.1016/s0378-1119(98)00131-0

[21] Paolini S, Scaloni A, Amoresano A, Marchese S, Napolitano E, et al (1998) Amino acid sequence post-translational modifications binding and labelling of porcine odorant-binding protein. Chem Senses 23: 689–698. 9915115 10.1093/chemse/23.6.689

[22] Ganni M, Garibotti M, Scaloni A, Pucci P, Pelosi P (1997) Microheterogeneity of odorant-binding proteins in the porcupine revealed by N-terminal sequencing and mass spectrometry. Comp Biochem Physiol 117B: 287–291. 10.1016/s0305-0491(97)00089-8 9226887

[23] Garibotti M, Navarrini A, Pisanelli AM, Pelosi P (1997) Three odorant-binding proteins from rabbit nasal mucosa. Chem Senses 22: 383–390. 9279461 10.1093/chemse/22.4.383

[24] Pes D, Pelosi P (1995) Odorant-binding proteins of the mouse. Comp Biochem Physiol 112B: 471–479. 10.1016/0305-0491(95)00063-1 8529023

[25] D'Innocenzo B, Salzano AM, D'Ambrosio C, Gazzano A, Niccolini A, et al (2006) Secretory proteins as potential semiochemical carriers in the horse. Biochemistry 45: 13418–13428. 17087495 10.1021/bi061409p

[26] Millery J, Briand L, Bezirard V, Blon F, Fenech C, et al (2005) Specific expression of olfactory binding protein in the aerial olfactory cavity of adult and developing *Xenopus* . Eur J Neurosci 22: 1389–1399. 16190893 10.1111/j.1460-9568.2005.04337.x

[27] Dal Monte M, Centini M, Anselmi C, Pelosi P (1993) Binding of selected odorants to bovine and porcine odorant binding proteins. Chem Senses 18: 713–721.

[28] Pevsner J, Hou V, Snowman AM, Snyder SH (1990) Odorant-binding protein characterization of ligand binding. J Biol Chem 265: 6118–6125. 2318850

[29] Hérent MF, Collin S, Pelosi P (1995) Affinities of nutty and green-smelling compounds to odorant-binding proteins. Chem Senses 20: 601–610. 8788094 10.1093/chemse/20.6.601

[30] Loebel D, Marchese S, Krieger J, Pelosi P, Breer H (1998) Subtypes of odorant binding proteins: heterologous expression and assessment of ligand binding. Eur J Biochem 254: 318–324. 9660186 10.1046/j.1432-1327.1998.2540318.x

[31] Vincent F, Ramoni R, Spinelli S, Grolli S, Tegoni M, et al (2004) Crystal structures of bovine odorant-binding protein in complex with odorant molecules. Eur J Biochem 271: 3832–3842. 15373829 10.1111/j.1432-1033.2004.04315.x

[32] Pelosi P (1998) Odorant-binding proteins: structural aspects. Ann NY Acad Sci 855: 281–293. 9929622 10.1111/j.1749-6632.1998.tb10584.x

[33] Pelosi P (2001) The role of perireceptor events in vertebrate olfaction. Cell Mol Life Sci 58: 503–509. 11361085 10.1007/PL00000875 PMC11146477

[34] Loebel D, Scaloni A, Paolini S, Fini C, Ferrara L, et al (2000) Cloning, post-translational modifications, heterologous expression, ligand-binding and modelling of boar salivary lipocalin. Biochem J 350: 369–379. 10947950 PMC1221263

[35] Pelosi P, Zhou J-J, Ban LP, Calvello M (2006) Soluble proteins in insect chemical communication. Cell Mol Life Sci 63: 1658–1676. 16786224 10.1007/s00018-005-5607-0 PMC11136032

[36] Xu P, Atkinson R, Jones DN, Smith DP (2005) *Drosophila* OBP LUSH is required for activity of pheromone-sensitive neurons. Neuron 45: 193–200. 15664171 10.1016/j.neuron.2004.12.031

[37] Matsuo T, Sugaya S, Yasukawa J, Aigaki T, Fuyama Y (2007) Odorant-binding proteins OBP57d and OBP57e affect taste perception and host-plant preference in *Drosophila sechellia* . PLoS Biol 5: e118. 17456006 10.1371/journal.pbio.0050118 PMC1854911

[38] Swarup S, Williams TI, Anholt RR (2011) Functional dissection of Odorant binding protein genes in *Drosophila melanogaster* . Genes Brain Behav 10: 648–657. 21605338 10.1111/j.1601-183X.2011.00704.x PMC3150612

[39] Sun YF, De Biasio F, Qiao HL, Iovinella I, Yang SX, et al (2012) Two Odorant-Binding Proteins Mediate the Behavioural Response of Aphids to the Alarm Pheromone (*E*)-ß-farnesene and Structural Analogues. PLoS One 7: e32759. 22427877 10.1371/journal.pone.0032759 PMC3299684

[40] Pevsner J, Hwang PM, Sklar PB, Venable JC, Snyder SH (1988) Odorant-binding protein and its mRNA are localized to lateral nasal gland implying a carrier function. Proc Natl Acad Sci USA 85: 2383–2387. 3353387 10.1073/pnas.85.7.2383 PMC279997

[41] Miyawaki A, Matsushita F, Ryo Y, Mikoshiba K (1994) Possible pheromone-carrier function of two lipocalin proteins in the vomeronasal organ. EMBO J 13: 5835–5842. 7813422 10.1002/j.1460-2075.1994.tb06927.x PMC395557

[42] Ohno K, Kawasaki Y, Kubo T, Tohyama M (1996) Differential expression of odorant-binding protein genes in rat nasal glands: implications for odorant-binding protein II as a possible pheromone transporter. Neuroscience 71: 355–366. 9053791 10.1016/0306-4522(95)00454-8

[43] Avanzini F, Bignetti E, Bordi C, Carfagna G, Cavaggioni A, et al.. (1987) Immunocytochemical localization of pyrazine-binding protein in bovine nasal mucosa. Cell Tissue Res 247: , 461–464. 10.1007/BF00218329 3545483

[44] Briand L, Eloit C, Nespoulous C, Bézirard V, Huet JC, et al.. (2002) Evidence of an odorant-binding protein in the human olfactory mucus: location, structural characterization, and odorant-binding properties. Biochemistry 41: , 7241–7252. 10.1021/bi015916c 12044155

[45] Cavaggioni A, Mucignat-Caretta C (2000) Major urinary proteins, alpha(2U)-globulins and aphrodisin. Biochim Biophys Acta 1482: 218–228. 11058763 10.1016/s0167-4838(00)00149-7

[46] Cavaggioni A, Findlay JB, Tirindelli R (1990) Ligand binding characteristics of homologous rat and mouse urinary proteins and pyrazine binding protein of calf. Comp Biochem Physiol B 96: 513–520. 2390861 10.1016/0305-0491(90)90049-y

[47] Robertson DHL, Beynon RJ, Evershed RP (1993) Extraction characterisation and binding analysis of two pheromonally active ligands associated with major urinary protein of the house mouse (*Mus musculus*). J Chem Ecol 19: 1405–1416. 24249171 10.1007/BF00984885

[48] Hurst JL, Payne CE, Nevison CM, Marie AD, Humphries RE, et al (2001) Individual recognition in mice mediated by major urinary proteins. Nature 414: 631–634. 11740558 10.1038/414631a

[49] Singer AG, Macrides F, Clancy AN, Agosta WC (1986) Purification and analysis of a proteinaceous aphrodisiac pheromone from hamster vaginal discharge. J Biol Chem 261: 13323–13326. 3759967

[50] Vincent F, Löbel D, Brown K, Spinelli S, Grote P, et al (2001) Crystal structure of aphrodisin, a sex pheromone from female hamster. J Mol Biol 305: 459–469. 11152604 10.1006/jmbi.2000.4241

[51] Scaloni A, Paolini S, Brandazza A, Fantacci M, Marchese S, et al (2001) Purification, cloning and characterisation of novel odorant-binding proteins in the pig. Cell Mol Life Sci 58: 823–834. 11437241 10.1007/PL00000903 PMC11337358

[52] Bacchini A, Gaetani E, Cavaggioni A (1992) Pheromone binding proteins in the mouse *Mus musculus* . Experientia 48: 419–421. 1374722 10.1007/BF01923448

[53] Rouvinen J, Rautiainen J, Virtanen T, Zeiler T, Kauppinen J, et al (1999) Probing the molecular basis of allergy Three-dimensional structure of the bovine lipocalin allergen Bos d2. J Biol Chem 274: 2337–2343. 9891000 10.1074/jbc.274.4.2337

[54] Hilger C, Kuehn A, Hentges F (2012) Animal lipocalin allergens. Curr Allergy Asthma Rep 12: 438–447. 22791068 10.1007/s11882-012-0283-2

[55] Mechref Y, Zidek L, Ma W-D, Novotny MV (2000) Glycosilated major urinary protein of the house mouse: characterization of its N-linked oligosaccharides. Glycobiology 10: 231–235. 10704521 10.1093/glycob/10.3.231

[56] Virtanen T, Kinnunen T, Rytkönen-Nissinen M (2012) Mammalian lipocalin allergens—insights into their enigmatic allergenicity. Clin Exp Allergy 42: 494–504. 22093088 10.1111/j.1365-2222.2011.03903.x

[57] Schaal B, Coureaud G, Langlois D, Giniès C, Sémon E, et al (2003) Chemical and behavioural characterization of the rabbit mammary pheromone. Nature 424: 68–72. 12840760 10.1038/nature01739

[58] Charra R, Datiche F, Casthano A, Gigot V, Schaal B, et al (2012) Brain processing of the mammary pheromone in newborn rabbits. Behav Brain Res 226: 179–188. 21925546 10.1016/j.bbr.2011.09.008

[59] Salzano AM, Novi G, Arioli S, Corona S, Mora D, et al (2013) Mono-dimensional blue native-PAGE and bi-dimensional blue native/urea-PAGE or/SDS-PAGE combined with nLC-ESI-LIT-MS/MS unveil membrane protein heteromeric and homomeric complexes in *Streptococcus thermophilus* . J Proteomics 94: 240–261. 24061001 10.1016/j.jprot.2013.09.007

[60] Scaloni A, Monti M, Angeli S, Pelosi P (1999) Structural analysis and disulfide-bridge pairing of two odorant-binding proteins from *Bombyx mori* . Biochem Biophys Res Comm 266: 386–391. 10600513 10.1006/bbrc.1999.1791

[61] Picariello G, Ferranti P, Mamone G, Roepstorff P, Addeo F (2008) Identification of N-linked glycoproteins in human milk by hydrophilic interaction liquid chromatography and mass spectrometry. Proteomics 8: 3833–3847. 18780401 10.1002/pmic.200701057

[62] Hilvo M, Baranauskiene L, Salzano AM, Scaloni A, Matulis D, et al (2008) Biochemical characterization of CA IX, one of the most active carbonic anhydrase isozymes. J Biol Chem 283: 27799–27809. 18703501 10.1074/jbc.M800938200

[63] Perez-Miller S, Zou Q, Novotny MV, Hurley TD (2010) High resolution X-ray structures of mouse major urinary protein nasal isoform in complex with pheromones. Protein Sci 19: 1469–1479. 20509168 10.1002/pro.426 PMC2923500

[64] Cavaggioni A, Sorbi RT, Keen JN, Pappin DJC, Findlay JBC (1987) Homology between the pyrazine-binding protein from nasal mucosa and major urinary proteins. FEBS Lett 212: 225–228. 3817156 10.1016/0014-5793(87)81349-2

[65] Finlayson JS, Asofsky R, Potter M, Runner CC (1965) Major urinary protein complex of normal mice: origin. Science 149: 981–982. 5827345 10.1126/science.149.3687.981

[66] Dinh BL, Tremblay A, Dufour D (1965) Immunochemical study of rat urinary proteins: their relation to serum and kidney proteins. J Immunol 95: 574–582. 4158465

[67] Zhang Z-D, Frankish A, Hunt T, Harrow J, Gerstein M (2010) Identification and analysis of unitary pseudogenes: historic and contemporary gene losses in humans and other primates. Genome Biology 11: R26. 20210993 10.1186/gb-2010-11-3-r26 PMC2864566

[68] Sun YL, Huang LQ, Pelosi P, Wang CZ (2012) Expression in antennae and reproductive organs suggests a dual role of an odorant-binding protein in two sibling *Helicoverpa* species. PLoS One 7: e30040. 22291900 10.1371/journal.pone.0030040 PMC3264552

[69] Lacazette E, Gachon A-M, Pitiot G (2000) A novel human odorant-binding protein gene family resulting from genomic duplicons at 9q34: differential expression in the oral and genital spheres. Hum Mol Genetics 9: 289–301. 10.1093/hmg/9.2.289 10607840

